# Molecular Docking and Molecular Dynamics Simulations in Related to *Leishmania donovani*: An Update and Literature Review

**DOI:** 10.3390/tropicalmed8100457

**Published:** 2023-09-26

**Authors:** Mabel R. Challapa-Mamani, Eduardo Tomás-Alvarado, Angela Espinoza-Baigorria, Darwin A. León-Figueroa, Ranjit Sah, Alfonso J. Rodriguez-Morales, Joshuan J. Barboza

**Affiliations:** 1 Escuela de Medicina, Universidad Cesar Vallejo, Trujillo 13007, Peru; machallapam@ucvvirtual.edu.pe; 2 Sociedad Científica de Estudiantes de Medicina de la Universidad César Vallejo, Trujillo 13007, Peru; 3 Hospital General Regional 17, Instituto Mexicano del Seguro Social, Cancún 75533, Mexico; acteck333@gmail.com; 4 Escuela de Medicina, Universidad Privada Antenor Orrego, Trujillo 13008, Peru; aespinozab2@upao.edu.pe; 5 Facultad de Medicina Humana, Universidad San Martín de Porres, Chiclayo 14006, Peru; darwin_leon@usmp.pe; 6 Department of Clinical Microbiology, Institute of Medicine, Tribhuvan University Teaching Hospital, Kathmandu 44600, Nepal; ranjitsah@iom.edu.np; 7 Department of Microbiology, Dr. D. Y. Patil Medical College, Hospital and Research Centre, Dr. D. Y. Patil Vidyapeeth, Pune 411018, Maharashtra, India; 8 Faculty of Health Sciences, Universidad Científica del Sur, Lima 150152, Peru; arodriguezmo@cientifica.edu.pe; 9 Gilbert and Rose-Marie Chagoury School of Medicine, Lebanese American University, Beirut 350000, Lebanon

**Keywords:** leishmaniasis, molecular docking, molecular dynamics (MD) simulation, therapeutic targets, genomic sequence

## Abstract

Leishmaniasis, a disease caused by *Leishmania* parasites and transmitted via sandflies, presents in two main forms: cutaneous and visceral, the latter being more severe. With 0.7 to 1 million new cases each year, primarily in Brazil, diagnosing remains challenging due to diverse disease manifestations. Traditionally, the identification of *Leishmania* species is inferred from clinical and epidemiological data. Advances in disease management depend on technological progress and the improvement of parasite identification programs. Current treatments, despite the high incidence, show limited efficacy due to factors like cost, toxicity, and lengthy regimens causing poor adherence and resistance development. Diagnostic techniques have improved but a significant gap remains between scientific progress and application in endemic areas. Complete genomic sequence knowledge of *Leishmania* allows for the identification of therapeutic targets. With the aid of computational tools, testing, searching, and detecting affinity in molecular docking are optimized, and strategies that assess advantages among different options are developed. The review focuses on the use of molecular docking and molecular dynamics (MD) simulation for drug development. It also discusses the limitations and advancements of current treatments, emphasizing the importance of new techniques in improving disease management.

## 1. Introduction

Leishmaniasis is a disease caused by different species of parasites of the genus *Leishmania*, with female sandflies as the transmission vector [1]. Various species of *Leishmania* produce different clinical manifestations, with two clinically notable forms: cutaneous and visceral. The visceral form is characterized by systemic involvement and greater severity [2].

Encompassing over 60 countries where it is considered an endemic disease, it is estimated that there are between 0.7 and 1 million new cases of leishmaniasis per year, with a global incidence of visceral leishmaniasis (VL) ranging from 50,000 to 90,000 reported in 2017. Brazil has the highest number of reported cases in Latin America, accounting for over 99% of the cases [3].

The clinical diagnosis of the disease is challenging due to the various manifestations and lesions that may appear depending on the spectrum and type of the disease [4]. Therefore, currently, the identification of different *Leishmania* species is mostly inferred from the clinical and epidemiological background [5].

Progress in the diagnosis and treatment of Leishmaniosis relies directly on the development of new technological tools and advancements that enable the creation and improvement of parasite identification and typing programs [6]. Despite the millions of cases detected each year, current treatments do not demonstrate sufficient efficacy to combat the disease.

A crucial point in laboratory diagnosis is the detection of the etiological agent and the identification of the species to which it belongs [7]. Despite the significant advances in diagnostic techniques, there are still significant differences between scientific advancements and diagnostic processes in *Leishmania* infections in endemic areas [4,8]. Therefore, the great challenge for researchers and clinicians is to bridge this gap and facilitate access to and understanding of this knowledge in the most affected areas [9].

Since the invention of techniques such as DNA amplification via PCR, there has been continuous improvement in the speed and effectiveness of *Leishmania* identification in biological samples [10].

Consequently, research and the search for other versatile and cost-effective methods for detecting pathogenic *Leishmania* species should be encouraged, allowing for a better understanding of genomic structure, genetic activity, protein expression, and metabolic changes that translate into improved diagnostic and therapeutic processes.

Thus, for example, knowledge of the complete genomic sequence of *Leishmania* increases the possibilities of identifying therapeutic targets [11].

The use of new computational tools allows for the identification of therapeutic targets and the development of strategies that assess advantages among the different available options, thus reducing both time and costs [12].

Different techniques such as virtual screening or molecular dynamics focus on optimizing processes for testing, searching, and detecting affinity in molecular docking; simulating biological systems; and even analyzing internal motions of proteins [13].

Molecular dynamics simulation is a computational method that provides information about the behavior of any molecular system by integrating Newton’s laws of motion, thus refining the protein structure of binding sites and providing relevant and useful information for understanding and comparing different interactions [13].

The use of techniques such as molecular docking and molecular dynamics simulation allows the development of more effective therapeutic strategies with greater advantages against *Leishmania* infection. Molecular dynamics simulations provide knowledge about the binding modes of inhibitors, identifying better strategies and more suitable drugs with fewer disadvantages and side effects for the treatment of leishmaniasis [13].

The goal of this review is to describe the foundations, key features, and significance of computational tools in the field of leishmaniasis. By initially providing a description of protein structure and function, it allows for an understanding of the development and applicability of techniques and algorithms, highlighting recent advancements, use cases, and limitations.

Our review primarily focuses on describing the necessary concepts of molecular docking and molecular dynamics (MD) simulation, highlighting their importance in the development of new drugs, as well as the potential and applicability of these techniques in current clinical settings in the field of leishmaniasis. We emphasize the different techniques and algorithms used for this purpose.

Furthermore, we also highlight the fact that it has become a significant health problem, discussing the advances and limitations of current treatment and the development of new techniques such as molecular docking and molecular dynamics (MD) simulation, which have become fundamental tools for a disease whose diagnosis has traditionally relied on parasite isolation, visualization, and cultivation in infected tissue.

## 2. Basic Concepts

### 2.1. Introduction to Protein Structure and Function

The fundamental macromolecules known as proteins are necessary for many biological functions [14]. For researchers to fully understand the molecular pathways underlying diseases like leishmaniasis, which is caused by protozoan parasites such as *Leishmania donovani*, a thorough understanding of their structure and function is essential. In this paper, we give a brief overview of protein structure and function in simulations of molecular dynamics and docking [15].

Amino acids are the building blocks of proteins, and peptide bonds bind them together. The primary structure of a protein is determined by its linear amino acid sequence [16]. The secondary, tertiary, and quaternary structures of the polypeptide chain are formed during folding, which also gives rise to the chain’s three-dimensional structure [17].

The polypeptide chain’s regional folding patterns, which largely involve α-helices and β-sheets, are called the secondary structure. Between the atoms in the backbone, hydrogen bonds hold these structural motifs in place [18]. The tertiary structure depicts the protein’s overall three-dimensional arrangement, including the spatial organization of its folds, loops, and secondary structure components. The stability of the tertiary structure is influenced by a number of non-covalent interactions, including van der Waals contacts, electrostatic forces, and hydrophobic interactions [19].

When proteins are made up of many subunits, the quaternary structure is important. Functional complexes are produced as a result of interactions between these subunits. Protein function and structure are closely related [20]. In addition to enzyme catalysis, signal transduction, molecular recognition, and structural support, proteins also perform a wide variety of other tasks [21].

The investigation of protein–ligand interactions and dynamic behavior makes use of the potent computational tools of molecular docking and molecular dynamics simulations. The binding style and affinities of a ligand to a protein are predicted by molecular docking [22]. It entails looking for energetically advantageous ligand conformations within the binding site of the protein. In order to rank candidate ligands and determine the binding free energy, various scoring functions are applied [23].

On the other hand, molecular dynamics simulations use conventional Newtonian dynamics methods to model the mobility and behavior of atoms over time. Investigating the dynamic behavior of proteins and their interactions with ligands involves solving the equations of motion [24]. Insights into protein flexibility, conformational changes, and stability, which are essential for comprehending their function and developing therapeutic interventions, can be obtained through simulations.

In the case of *Leishmania donovani*, protein–ligand interactions crucial to the parasite’s survival and growth have been examined using molecular docking and molecular dynamics simulations [25]. These computational techniques have contributed to the discovery of possible therapeutic targets, the creation of fresh inhibitors, and the enhancement of currently available medications. They have also provided important information for drug discovery efforts by shedding light on the dynamics and structural changes of crucial proteins [26].

### 2.2. Description of Molecular Docking

A computer method that is essential in the development of new drugs is molecular docking. Through an examination of their potential conformations and interactions, it entails predicting the optimal orientation and binding affinity of a tiny molecule (ligand) to a target protein (receptor) [27]. The parasite *Leishmania donovani* causes the neglected tropical disease VL, and molecular docking has been proven to be a useful technique for locating prospective therapeutic targets, creating new medications, and improving already-effective therapies [28].

#### 2.2.1. Primary Ideas

The fundamental concepts of molecular recognition and binding interactions serve as the foundation for molecular docking. Predicting a ligand’s binding mechanism in a receptor’s active region and calculating the binding affinity are the main goals. Important ideas include the following:

1. *Molecular Representation*: Ligands and receptors are frequently depicted as three-dimensional structures with atomic characteristics, such as charge and atom kinds, that are connected to them [29].
2. *Search Algorithms*: To explore the conformational space of the ligand and receptor and find the best binding mode, docking algorithms use a variety of search methodologies. To assess the energy or fitness of each conformation, these algorithms make use of scoring functions [30].
3. *Scoring Functions*: By taking into account variables including van der Waals interactions, electrostatic interactions, hydrogen bonds, and solvation effects, scoring functions calculate the binding affinity between a ligand and receptor [31]. The scoring function assigns a numerical value to binding affinity and rates various ligand postures.

#### 2.2.2. Used Techniques and Algorithms

For molecular docking, numerous techniques and algorithms have been developed. Several of the commonly employed methods include the following:

1. *Structure-based Docking*: This technique makes use of the ligand and receptor’s three-dimensional structures [30]. Using search algorithms like Genetic Algorithms, Monte Carlo techniques, or molecular dynamics simulations, it investigates the conformational space of the ligand and receptor to identify the most advantageous binding mode [22].
2. *Ligand-based Docking*: When the receptor structure is unknown, ligand-based docking techniques are applied. These techniques are dependent on understanding the known ligands that bind to the receptor [32]. To find candidate ligands with related features, similarity-based methods like virtual screening and pharmacophore modeling are applied [33].

#### 2.2.3. Applications in the Field of Leishmaniasis

Molecular docking has significantly aided efforts to find new treatments for leishmaniasis. A few noteworthy applications are as follows:

1. *Target Identification*: In order to discover possible therapeutic targets in *Leishmania donovani*, docking experiments have been used. Researchers can find important proteins involved in the parasite’s survival and growth by examining the interactions between known ligands and target proteins.
2. *Virtual Screening*: Docking-based virtual screening makes it possible to quickly screen sizable chemical libraries for prospective *Leishmania donovani* therapeutic candidates. Researchers might give higher priority to substances with strong binding affinity for additional experimental validation by docking small molecules against target proteins.
3. *Drug Design and Optimization*: Docking is essential to the logical development and refinement of medications to combat *Leishmania donovani*. To increase the potency, selectivity, and pharmacokinetic features of lead drugs, researchers can change and enhance the binding relationships between ligands and target proteins.

## 3. Molecular Docking Applications in Leishmaniasis

### 3.1. Selection of Therapeutic Targets

Molecular docking assists in the selection of suitable therapeutic targets in the *Leishmania* parasite. By analyzing the protein structures and their interactions with ligands, docking simulations can identify essential proteins involved in the parasite’s survival and virulence. This information aids in the prioritization of potential targets for drug intervention.

Molecular docking has played a crucial role in the identification and selection of therapeutic targets for Leishmaniasis [34]. This approach enables researchers to predict the binding affinity and mode of interaction between small molecules and specific target proteins within the *Leishmania* parasite. By understanding the molecular interactions, researchers can prioritize and select potential therapeutic targets for further investigation [35]. The selection of therapeutic targets in Leishmaniasis often focuses on proteins that are vital for the parasite’s survival, growth, or virulence. These proteins may include enzymes involved in metabolic pathways, transporters, surface receptors, or signaling proteins [36]. By targeting these essential proteins, it becomes possible to disrupt crucial biological processes in the parasite, leading to its inhibition or elimination. Molecular docking studies aid in the identification of potential target proteins by screening large databases of protein structures. Computational tools can analyze the binding sites of these proteins and predict their suitability as drug targets based on factors such as druggability, accessibility, and essentiality for the parasite’s survival. The docking simulations can evaluate the binding affinity and specificity of small molecules toward these target proteins [37]. Additionally, molecular docking helps prioritize potential drug candidates by assessing their binding affinities and interactions with the selected target proteins. Compounds with favorable docking scores and strong binding interactions can be considered promising leads for further experimental validation [38].

It is worth noting that the selection of therapeutic targets in Leishmaniasis is a complex process that requires a comprehensive understanding of the parasite’s biology and pathogenesis. The integration of experimental data, bioinformatics analysis, and structural biology techniques can complement molecular docking studies and provide a more holistic approach to target selection. In conclusion, molecular docking plays a significant role in the selection of therapeutic targets for Leishmaniasis. By identifying and prioritizing proteins that are crucial for the parasite’s survival, researchers can focus their efforts on developing effective treatments to combat this disease [39].

### 3.2. Identification of Inhibitors and Candidate Molecules

Molecular docking enables the screening and identification of inhibitors and candidate molecules that can interact with the selected targets [40]. Virtual docking experiments simulate the binding interactions between small molecule compounds or potential drugs and the target proteins. By analyzing the binding affinities and interaction patterns, researchers can identify compounds with high binding affinity and favorable drug-like properties. These identified molecules can serve as starting points for further development and optimization into potential drugs for leishmaniasis treatment [41].

One of the primary goals of molecular docking in Leishmaniasis research is to identify small molecules that can act as inhibitors of essential proteins or enzymes involved in the parasite’s survival and replication [41]. By targeting these proteins, researchers aim to disrupt critical biological processes and inhibit the growth and proliferation of the parasite. In the initial stage of the molecular docking process, researchers build a library of small molecules or obtain one from existing databases [42]. These compounds can be natural products, synthetic chemicals, or repurposed drugs. The compounds are virtually screened against a selected target protein, and docking simulations are performed to predict their binding affinity and mode of interaction. The docking scores obtained from these simulations help prioritize and rank the potential inhibitors. Compounds with favorable docking scores and strong interactions with the target protein are considered promising candidates for further evaluation. These candidate molecules can then be subjected to experimental validation, such as in vitro assays or animal models, to assess their efficacy and specificity against *Leishmania* parasites [43].

Moreover, molecular docking can aid in the optimization of lead compounds by exploring modifications and predicting their binding affinities. This process, known as structure-based drug design, allows researchers to make chemical modifications to the identified hit compounds, enhancing their potency, selectivity, and pharmacokinetic properties. Iterative docking simulations can guide the design and synthesis of analogs or derivatives with improved inhibitory activity [44].

In recent years, advances in computational methods, increased availability of protein structures, and compound libraries have contributed to the identification of potential inhibitors and candidate molecules against Leishmaniasis. Molecular docking has been proven to be a valuable approach for the initial screening and prioritization of compounds, accelerating the drug discovery process for this disease [44].

### 3.3. Evaluation of the Activity of Existing Drugs

Molecular docking also plays a vital role in evaluating the activity of existing drugs against leishmaniasis. By docking approved drugs or known compounds against specific target proteins, researchers can assess their binding affinities and predict their efficacy in inhibiting the target’s function [45]. This approach provides valuable insights into repurposing existing drugs for leishmaniasis treatment, potentially accelerating the drug discovery process by identifying candidates that can be readily tested in preclinical and clinical studies [46].

The evaluation of existing drugs in Leishmaniasis through molecular docking involves the following steps:

1. Selection of Target Proteins: Specific proteins that play crucial roles in the *Leishmania* parasite’s lifecycle or pathogenesis are identified as potential targets. These proteins may include enzymes, transporters, receptors, or other key molecules involved in essential biological processes [47].
2. Building Compound Libraries: A library of known drugs is compiled, including approved drugs, experimental compounds, or compounds from existing databases. This diverse collection of molecules serves as a resource for virtual screening and docking simulations [47].
3. Virtual Screening: Virtual screening involves the computational docking of the compounds from the library onto the target proteins. The docking algorithms predict the binding orientations and affinities of the drugs within the protein’s active site, allowing for the identification of potential drug–protein interactions [47].
4. Binding Affinity Analysis: The docking scores obtained from the simulations provide a measure of the binding affinity between the drugs and the target proteins. Compounds with high docking scores are considered to have strong binding potential and are further investigated for their activity against *Leishmania* parasites [47].
5. Interaction Analysis: The docking results are analyzed to understand the specific interactions between the drugs and the target proteins. This analysis helps to identify key molecular interactions, such as hydrogen bonding, hydrophobic interactions, or electrostatic interactions, that contribute to the binding and potential inhibitory activity [47].
6. Experimental Validation: Promising drug candidates identified through molecular docking are subjected to experimental validation to assess their activity against *Leishmania* parasites. In vitro assays, such as enzyme inhibition assays or parasite growth inhibition assays, help determine the efficacy and selectivity of the drugs. Animal models may also be used to evaluate the in vivo activity and toxicity profiles of the drug candidates [47].
7. Optimization and Lead Refinement: Based on the results of molecular docking and experimental validation, lead compounds can be optimized through structure-based drug design approaches. Iterative docking simulations and computational chemistry techniques assist in modifying the chemical structures of the drug candidates to improve their potency, selectivity, and pharmacokinetic properties [47].

By employing molecular docking in the evaluation of existing drugs, researchers can expedite the drug discovery process for Leishmaniasis. This approach offers the advantage of repurposing known drugs, which have already undergone safety and toxicity assessments, potentially reducing the time and cost required for drug development [48].

Overall, molecular docking is a valuable tool in the field of leishmaniasis research and drug discovery. It aids in the selection of therapeutic targets, the identification of inhibitors and candidate molecules, and the evaluation of existing drugs. By leveraging the computational power of molecular docking, researchers can expedite the search for effective treatments against leishmaniasis, potentially leading to the development of novel therapeutics and improved outcomes for patients affected by this global health problem [48].

## 4. Applications of Molecular Dynamics Simulations in Leishmaniasis

Molecular dynamics (MD) simulations are computational techniques used to study the behavior and dynamics of biological molecules at an atomic level [49]. In the context of leishmaniasis, MD simulations have several applications that aid in understanding protein–ligand interactions, stability, conformational studies, and the analysis of conformational and dynamic changes in proteins. Below is an elaboration on these applications.

### 4.1. Characterization of Protein–Ligand Interactions

MD simulations provide insights into the binding mechanisms and dynamics of protein–ligand interactions in leishmaniasis. By simulating the movement of the protein and ligand over time, researchers can observe the formation and breaking of hydrogen bonds, van der Waals interactions, and other non-covalent interactions that contribute to binding [50]. This information helps in understanding the strength and stability of the protein–ligand complex and guides the design of more potent and selective inhibitors.

### 4.2. Stability and Conformational Studies

MD simulations are valuable for investigating the stability and conformational changes of proteins involved in leishmaniasis. By subjecting the protein to a simulated environment, researchers can observe its behavior, folding, and unfolding over time [51]. This allows the assessment of stability, identification of key regions responsible for structural changes, and exploration of the effects of mutations or ligand binding on protein dynamics. Such insights are crucial for understanding the functional properties of proteins and can aid in drug design and optimization [52,53].

### 4.3. Analysis of Conformational and Dynamic Changes in Proteins

MD simulations provide a detailed view of the conformational and dynamic changes that occur in proteins relevant to leishmaniasis. By analyzing trajectories generated from MD simulations, researchers can identify important structural transitions, such as loop movements, domain motions, or protein unfolding [53,54]. This knowledge can uncover key functional regions or reveal allosteric sites that can be targeted for drug intervention. Additionally, comparing MD simulations of different protein states can shed light on the conformational changes associated with protein function and provide insights into potential drug-binding sites [55].

## 5. Recent Advances and Case Studies

### 5.1. Review of Recent Studies Using Molecular Docking and Molecular Dynamics Simulations in Leishmaniasis

In recent years, there have been notable advancements in the application of molecular docking and molecular dynamics (MD) simulations in the field of leishmaniasis research. Several studies have utilized these computational techniques to investigate protein–ligand interactions, protein dynamics, and drug discovery [56]. These studies have contributed valuable insights into the molecular mechanisms underlying leishmaniasis and have aided in the identification of potential therapeutic targets and drug candidates.

One recent study utilized molecular docking to screen a library of natural compounds against leishmanial proteins [57]. The results identified several promising compounds with high binding affinities, suggesting their potential as antileishmanial agents. Another study employed MD simulations to investigate the stability and dynamics of drug-resistant forms of leishmanial proteins. The simulations revealed conformational changes associated with resistance, providing crucial information for designing new inhibitors to overcome drug resistance [58].

Computational research is currently underway to identify potential small molecule inhibitors targeting *Leishmania* pteridine reductase 1 (PTR1), a crucial enzyme in parasite DNA synthesis [59]. The discovery of inhibitors for this enzyme is an active area of research. A recent study has revealed the existence of several compounds with a high binding affinity for PTR1, surpassing even the enzyme’s natural substrate. These interactions occur with specific residues in PTR1 that play an important role in its catalytic activity. Moreover, remarkable stability in the complexes formed between these compounds and PTR1 has been observed during computational simulations [60].

Another study used a computational approach to model the three-dimensional structure of ornithine decarboxylase from *Leishmania donovani* (Ld ODC), and high-throughput virtual screening was performed with 8630 ligands from the ZINC database [2]. Of these, 45 ligands were selected based on their high binding score and validated by molecular oxygen simulations. Two major molecules, ceftaroline fosamyl and rimegepant, were identified as the most promising and were subjected to molecular dynamics simulations, density functional theory, and generalized natal surface area analysis of molecular mechanics, and their results revealed that ceftaroline fosamyl and rimegepant exhibited binding affinities of −10,719 and −10,159 kcal/mol, respectively [2]. These in silico findings suggest that both ceftaroline fosamyl and rimegepant may be promising options for the development of treatments against leishmaniasis. However, further studies are required to evaluate and confirm their efficacy and safety [2].

In addition, recently, research has been conducted using molecular polymer techniques to predict protein–ligand interactions of both natural and human-made substances [61]. In particular, it has been found that *Withania somnifera*, an important medicinal plant in India, can be used in combination with effective doses of antileishmanial drugs as a promising alternative to treat VL, thereby enhancing the host immune response [62]. On this basis, recent research using the molecular antibody technique to predict protein–small molecule interactions has identified N-myristoyltransferase (NMT) as a promising therapeutic target for the development of new drugs and observed that natural compounds from *Withania somnifera*, such as calycopteretin-3-ruthinoside and withanoside IX, have been found to have a high affinity for *Leishmania* NMT and could be used as potential drugs or to design more effective inhibitors [41].

### 5.2. Relevant Results and Conclusions Drawn

The application of molecular docking and MD simulations in leishmaniasis research has yielded significant results and drawn important conclusions. These studies have identified potential therapeutic targets, such as essential leishmanial proteins involved in key biological processes, allowing researchers to design specific inhibitors to disrupt the parasite’s survival and proliferation [63].

Moreover, through molecular docking and MD simulations, researchers have identified lead compounds with high binding affinities to these targets, serving as potential candidates for drug development [64]. The computational techniques have also provided insights into the structural dynamics and stability of leishmanial proteins, aiding in the design of more effective drugs by targeting specific regions or allosteric sites.

Additionally, these studies have deepened our understanding of the interaction between leishmanial proteins and the host immune system. By elucidating the mechanisms of immune evasion, researchers can develop strategies to boost the host immune response and enhance the efficacy of immunotherapies [65].

Computational tools, such as molecular docking and molecular dynamics simulations, have emerged as indispensable techniques in the field of drug discovery for Leishmaniasis. These methods enable the investigation of protein–ligand interactions and provide valuable insights into the molecular mechanisms underlying the disease [66]. Through molecular docking, researchers can predict the binding affinity and orientation of potential drug candidates to target proteins involved in *Leishmania* infection. This information aids in the identification of novel therapeutic targets and the design of optimized drugs with enhanced efficacy and specificity [67].

Molecular dynamics simulations have proven instrumental in understanding the dynamic behavior of proteins and their interaction with ligands. By employing Newtonian dynamics methods, researchers can explore the conformational changes, flexibility, and stability of proteins associated with Leishmaniasis. This knowledge enhances our understanding of the disease mechanisms and assists in the rational design of targeted therapeutics [48].

The integration of molecular docking and molecular dynamics simulations has yielded significant advancements in Leishmaniasis research. These computational approaches have contributed to the identification of potential drug targets, the discovery of new inhibitors, and the optimization of existing drugs. By elucidating the structural and functional properties of protein targets, computational tools enable researchers to develop more effective treatments for Leishmaniasis [46].

The use of computational techniques not only accelerates the drug discovery process but also offers cost-effective alternatives to traditional experimental methods [68]. By reducing the reliance on laborious and expensive laboratory experiments, computational approaches provide a valuable avenue for screening large compound libraries and prioritizing potential drug candidates for further evaluation [46].

In summary, the integration of molecular docking and molecular dynamics simulations has revolutionized Leishmaniasis research by facilitating the identification and optimization of potential therapeutic targets. These computational tools have enhanced our understanding of the disease mechanisms and accelerated the development of effective treatments for this neglected tropical disease.

## 6. Limitations and Challenges

### 6.1. Description of Current Limitations in the Use of Molecular Docking and Molecular Dynamics Simulations in Leishmaniasis

While molecular docking and molecular dynamics (MD) simulations have become valuable tools in leishmaniasis research, there are certain limitations that need to be addressed. Some of the current limitations are described below.

Lack of accurate protein structures: Accurate and experimentally determined protein structures are essential for reliable molecular docking and MD simulations. However, the availability of crystal structures or high-quality protein models for leishmanial proteins can be limited, posing a challenge in accurately predicting protein–ligand interactions.

Challenges in simulating large protein systems: *Leishmania* proteins, especially those involved in complex biological processes, can be large and exhibit conformational flexibility. Simulating such large protein systems using MD simulations can be computationally demanding and time-consuming, limiting the scale and scope of the simulations.

Representation of water and solvation effects: Accurately representing the water molecules and solvation effects in molecular docking and MD simulations is crucial for capturing realistic protein–ligand interactions. However, the treatment of water molecules in these simulations often involves approximations, which can affect the accuracy of the results.

Force field limitations: Force fields, which describe the interactions between atoms and molecules in simulations, have inherent limitations. Force fields used in MD simulations may not fully capture the complexity of protein–ligand interactions, leading to inaccuracies in predicting binding affinities and protein dynamics.

### 6.2. Future Challenges and Promising Areas of Research

Despite the current limitations, there are promising areas of research and future challenges that can advance the use of molecular docking and MD simulations in leishmaniasis:

Improved accuracy in protein structure determination: Advances in experimental techniques, such as X-ray crystallography, cryo-electron microscopy, and nuclear magnetic resonance, will contribute to a more comprehensive understanding of leishmanial protein structures. This will enhance the accuracy of molecular docking and MD simulations [69].

Development of better force fields: Ongoing efforts to refine force fields and develop specialized parameters for leishmanial proteins will improve the accuracy of simulations and enable more accurate predictions of protein–ligand interactions.

Integration of multi-scale modeling: Combining molecular docking and MD simulations with other computational techniques, such as quantum mechanics or coarse-grained modeling, can provide a more comprehensive understanding of the dynamic behavior of leishmanial proteins and their interactions with ligands [69].

Incorporation of machine learning and artificial intelligence: The integration of machine learning and artificial intelligence algorithms can enhance the efficiency and accuracy of molecular docking and MD simulations [69]. These approaches can assist in predicting binding affinities, analyzing large datasets, and guiding the design of novel inhibitors.

## 7. Conclusions

In conclusion, while molecular docking and MD simulations have limitations in their current applications in leishmaniasis research, ongoing research efforts hold promise for addressing these challenges. Advances in protein structure determination, force field development, multi-scale modeling, and the incorporation of machine learning can improve the accuracy and applicability of these computational techniques, leading to a deeper understanding of leishmaniasis and the development of more effective treatments.

## Data Availability

Not applicable.
